# Isolation and Characterization of Twelve Polymorphic Microsatellite Loci for the Cocoa Mirid Bug *Sahlbergella Singularis*

**DOI:** 10.3390/ijms13044412

**Published:** 2012-04-10

**Authors:** Régis Babin, Catherine Fenouillet, Thierry Legavre, Laurence Blondin, Caroline Calatayud, Ange-Marie Risterucci, Marie-Pierre Chapuis

**Affiliations:** 1 Centre de Coopération Internationale en Recherche Agronomique pour le Développement (CIRAD), Unité Propre de Recherche Bioagresseurs Analyse et Maîtrise du Risque, F-34398 Montpellier, France; E-Mails: catherine.fenouillet@cirad.fr (C.F.); laurence.blondin@cirad.fr (L.B.); marie-pierre.chapuis@cirad.fr (M.-P.C.); 2 Centre de Coopération Internationale en Recherche Agronomique pour le Développement (CIRAD), Unité Mixte de Recherche Amélioration Génétique et Adaptation des Plantes Méditerranéennes et Tropicales, F-34398 Montpellier, France; E-Mails: thierry.legavre@cirad.fr (T.L.); caroline.calatayud@cirad.fr (C.C.); ange-marie.risterucci@cirad.fr (A.-M.R.)

**Keywords:** genetic markers, Miridae, *Sahlbergella singularis*, *Distantiella theobroma*, Bryocorinae

## Abstract

Mirids are the primary pests affecting cocoa production in Africa, but no genetic studies have been conducted on these insects. Here we report the isolation and characterization of 12 polymorphic microsatellite loci for *Sahlbergella singularis*. A microsatellite-enriched genomic DNA library was developed and screened to identify marker loci. Twelve polymorphic loci were identified by screening 28 individuals collected from one presumed population in cocoa plantations in Southern Cameroon. The number of alleles ranged from 5 to 25, whereas the observed and the expected heterozygosities ranged from 0.179 to 0.786 and from 0.671 to 0.946, respectively. Tests showed significant deviations from HW equilibrium for four loci, but no linkage disequilibrium was detected at any of the loci. No cross-species amplification was observed in two other mirid pests in Africa.

## 1. Introduction

The mirid *Sahlbergella singularis* Hagl. (Hemiptera: Miridae: Bryocorinae) is one of the primary pests affecting cocoa (*Theobroma cacao* L.) production in Africa, associated with 25 to 40% production losses. *Sahlbergella singularis* is widely distributed in West Africa, present throughout the forest zone, from Sierra Leone to the Demographic Republic of Congo [1], and its life history is well known on cocoa [1,2]. However, knowledge of *S. singularis* population structure in cocoa plantations is incomplete. About one century ago, mirids adapted to cocoa, a newly introduced cash-crop in West Africa. Its natural host-plants are mainly forest trees of the Malvaceae [1]. Although these trees are frequent neighbors or shade trees in cocoa agroforestry systems [3], their role in mirid population dynamics is unknown.

Molecular genetic techniques provide powerful tools for the study of insect ecology and population genetics in natural environment. DNA fingerprinting using microsatellite loci provides information on genetic variation at the population level allowing identification of biotypes and characterization of population structure, gene flow, and dispersal [4]. The present paper reports the isolation and characterization of microsatellite markers for *Sahlbergella singularis*. We also tested cross-species amplification for the closely related species *Distantiella theobroma* Distant. (Hemiptera: Miridae), the second most important pest of cocoa in West Africa [1], and for a mirid bug of sorghum, *Eurystylus oldi* Poppius (Hemiptera: Miridae).

## 2. Results and Discussion

Twelve primer pairs out of 28 tested showed a good amplification pattern and polymorphism in the 28 *S. singularis* individuals sampled from one presumed population for the study. The number of alleles per locus ranged from 5 to 25, while the observed and the expected heterozygosities ranged from 0.179 to 0.786 and from 0.671 to 0.946, respectively (Table 1). LD tests showed that there was no significant genetic association for all the 66 pairwise combinations of loci. The test showed significant deviations from HW equilibrium (*P* < 0.05) for four loci (Ss14, Ss01, Ss4 and Ss10). Analysis performed with MICRO-CHECKER showed that the general deficit in heterozygotes was distributed across most allele size classes excluding genotyping errors as a source of deviation from HW equilibrium. Our results showed that a significant excess of homozygotes for loci Ss14 and Ss10 might be due to high prevalence of null alleles (*i.e*., ≥20%; Table 1). Other loci, including Ss01, showed null allele frequencies ≤10% but Ss4 displayed a moderate frequency of 11%. The primary cause of null alleles is mutations in the primer-binding region flanking microsatellite sequences [5,6]. Null alleles are common in insects and similar studies for other mirid species also indicated signs of null alleles at several loci [7–9].

None of the *S. singularis* microsatellite primers used in this study amplified in either of the other two mirid species, which suggests that the primers designed for *S. singularis* in our study may be species-specific, with limited cross-species applicability.

## 3. Experimental Section

### 3.1. Isolation of Microsatellite Markers

Genomic DNA was extracted based on Risterucci *et al*. [10] from 5 pooled individuals (3 adults and 2 nymphs) collected on a cocoa farm in the Centre Region of Cameroon near Yaoundé (3°52′N; 11°28′E), and conserved in alcohol. A microsatellite-enriched genomic DNA library was developed following the method of Billotte *et al.* [11]: total genomic DNA was digested with the *Rsa*I. DNA fragments were purified and ligated with T4 DNA ligase (Gibco-BRL) to *Mlu*I self-complementary adaptators (RSA21 5′-CTCTTGCTTACGCGTGGACTA-3′ and phosphorylated RSA25 5′-TAGTCCACGCGTAAGCAAGAGCACA-3′). The selection of microsatellite sequences was performed following a hybridization-based capture method using biotin-labeled microsatellite oligoprobes and streptavidin-coated magnetic beads. The selected fragments were amplified using RSA21 primer. The resulting amplification products were cloned into pGEM-T Easy vector (Promega, Madison, USA), and transformed using *Epicurian coli* XL1-Blue MRF super-competent cells (Stratagene, www.genomics.agilent.com). One hundred ninety-two recombinant colonies were amplified with RSA21 primer. The size of inserts was estimated using agarose gel electrophoresis of PCR products. The electrophoresed PCR products were alkaline-Southern transferred onto Hybond N+ nylon membranes (Amersham, www.gelifesciences.com). Clones containing microsatellite alleles were selected by hybridization with a ^32^P radiolabeled (GA)_15_ and (GT)_15_ synthetic microsatellite probe. We sequenced the 93 recombinant clones that showed a strong hybridization signal and the SSR Analysis Tool (SAT) [12] was used to collect sequence information and facilitate the design of PCR primers and evaluation of flanking sequences. The following criteria were considered for sequence selection: uniqueness, adequate flanking sequence size, and a lack of repetitive elements in the flanking regions. Final primer pairs were designed using Primer 3 [13].

### 3.2. Primer Validation

Levels of locus polymorphism were assessed in 28 *S. singularis* individuals, sampled from a presumed population on cocoa, in a restricted area of the Bakoa village (4°34.3′N; 11°10.0′E) in the Centre Region of Cameroon. Since evidence was produced that individuals on a cocoa tree may be related [2], we collected a single individual per tree. PCR amplification was performed with a thermocycler TC-512 (Techne) following a Touchdown procedure [14] with an annealing temperature decrease of 0.5 °C per cycle during the first 10 cycles of the PCR (from 60 °C to 55 °C). PCR started with an initial activation step at 95 °C for 15 min followed by 35 cycles with denaturation at 94 °C for 30 s, annealing for 90 s, extension at 72 °C for 75 s and a 20 min final extension step at 72 °C.

Loci were combined in sets which were independently co-amplified using a multilocus amplification Kit (Qiagen) in a 10 μL volume containing 1× Qiagen Multiplex Master Mix (+Q), 0.2 μM of each primer and 2 μL of genomic DNA (20 ng/μL). Forward primers were labeled with FAM, VIC, or NED fluorescent labels. Labeled fragments were then discriminated using a capillary sequencer ABI 3500 (Applied Biosystems) with the size standard GeneScan 500 Liz. Allele sizes were determined using GENEMAPPER version 4.1 (Applied Biosystems).

Levels of expected and observed heterozygosities were computed using GENECLASS2 version 2.0.h [15]. Hardy–Weinberg (HW) tests for each locus and linkage disequilibrium (LD) tests for each pair of loci were performed using GENEPOP version 4.1 [16]. *P*-values of HW and LD tests were corrected with the sequential Bonferroni adjustment [17]. Mean null allele frequencies were computed using FREENA [6]. The most probable causes of deviation from HW equilibrium were determined among various genotyping errors and the presence of null alleles with MICRO-CHECKER version 2.2.3 [18].

### 3.3. Cross-Species Transferability

Cross-species amplification was attempted for 7 individuals of *Distantiella theobroma*, collected on *Bombax* sp. (Malvaceae) near Bokito (4°30.0′N; 11°04.8′E), and 6 individuals of *Eurystylus oldi*, collected on sorghum at Niamey, Niger (13°30.8′N; 2°06.7′E). Extraction and PCR amplification were performed as described for *S. singularis*.

## 4. Conclusions

To the best of our knowledge, no microsatellite markers have been developed for African cocoa mirids. A recent publication reports the description of 6 polymorphic loci for the polyphagous mirid Bryocorinae *Helopeltis theivora* Waterhous, a pest of cocoa in Asia [19]. Here we described twelve microsatellite loci for potential use in genetics studies of *Sahlbergella singularis* populations. Our work will improve the management of this important pest that affects cocoa production in Africa through better knowledge of mirid ecology in cocoa plantations. Conservation of primer sequences was not observed for the two other mirid species tested.

## Figures and Tables

**Table 1:** Twelve microsatellite loci isolated in *Sahlbergella singularis* collected from Southern Cameroon: locus name, PCR multiplex set no., repeat motif and allele size for the cloned allele, allele size range, number of alleles (*N*_a_), observed (*H*_o_) and expected (*H*_e_) heterozygosities, mean null allele frequency (*r*_null_), primer sequences and GenBank Accession no.

Locus	PCR multiplex set	Repeat motif	Allele size (bp)	Allele size range (bp)	*N*_a_	*H*_o_	*H*_e_	*r*_null_ (%)	Primer sequence (5′-3′)	Genbank Accession *N*°
Ss14	1	(GA)_30_CA(GA)_2_ AA(GA)_3_	234	205–278	16	0.462	0.917 *	23	F: *Pet*-CTGGAAATGGGTAGGGGATT R: GACAGGGTAGTCGGCAAGAC	JQ687207
Ss24	1	(AC)_26_	188	153–247	17	0.720	0.904	9	F: *Ned*-AAACACGACTTTTCCCTTAC R: AGCTAAAATGCTATCTCTGC	JQ687206
Ss01	2	(TC)_19_	239	216–252	18	0.750	0.927 *	7	F: *Fam*-TCCGAGGGAAACCTTCCTAT R: ACGTTATGCAGCACCGATTA	JQ687216
Ss11	2	(AC)_24_	155	119–151	12	0.750	0.871	4	F: *Fam*-GTCCATGCGAGCTGATGTT R: CGTCTCTCCTGCTTCATACG	JQ687213
Ss15	2	(GA)_28_	161	125–194	25	0.741	0.946	10	F: *Pet*-CGAAGCCAAGCGTATATTCC R: TGCGAGGTCGATAGTTTGAA	JQ687208
Ss04	3	(CT)_6_AT(CT)_7_ (CA)_3_CG(CA)_6_	217	187–234	17	0.643	0.851 *	11	F: *Fam*-*GGATGTTCCTTTACCGCTTT* R: ACATGAATAGCGTGAGATTCC	JQ687210
Ss05	3	(GT)_11_	120	104–125	9	0.714	0.872	8	F: *Fam*-CTAGTGATGGTATGTAATCAGC R: GTGAACTCTACAAGGGATAATG	JQ687217
Ss12	3	(AC)_14_	198	183–229	10	0.609	0.788	10	F: *Vic*-ACAACCAAGCTGATGTTTCG R: TCATTCATTACAGTGCCTCTTG	JQ687214
Ss06	4	(CA)_6_	100	96–102	5	0.536	0.671	4	F: *Vic*-TATAGGGCCAGGGGTAGACA R: AAAGGGCTGTAATCGAAATGC	JQ687215
Ss19	4	GACGAG(GA)_17_ GG(GA)_2_	160	130–183	14	0.786	0.865	4	F: *Vic-*CAGCAATGTCTTAATGTTCGAC R: TTGAAGCAGTGGCTCTTAATG	JQ687211
Ss10	na	(GT)_15_(GTGA)_3_ (GAAT)_3_	101	76–127	8	0.179	0.706 *	31	F: *Fam-*GCTGGGTATTTGAGAGGGATT R: CGCCAGATGAATAATAAAGACG	JQ687212
Ss25	na	(AC)_27_	231	191–231	13	0.778	0.861	4	F:*Fam*CGTTATCAGTATCATTCGAGCAGT R: GTTAGTCCTCGCCGCATCT	JQ687209

* indicates significant deviations from Hardy-Weinberg equilibrium after sequential Bonferroni correction (*p* < 0.05).

## References

[1] Entwistle P.F. (1972). Pests of Cocoa.

[2] Babin R., Anikwe J.C., Dibog L., Lumaret J.-P. (2011). Effects of cocoa tree phenology and canopy microclimate on the performance of the mirid bug *Sahlbergella singularis*. Entomol. Exp. Appl.

[3] Babin R., ten Hoopen G.M., Cilas C., Enjalric F., Yede, Gendre P., Lumaret J.-P. (2010). Impact of shade on the spatial distribution of *Sahlbergella singularis* in traditional cocoa agroforests. Agric. For. Entomol.

[4] Hoy M.A. (2003). Insect Molecular Genetics, an Introduction to Principles and Applications.

[5] Dakin E.E., Avise J.C. (2004). Microsatellite null alleles in parentage analysis. Heredity.

[6] Chapuis M.-P., Estoup A. (2007). Microsatellite null alleles and estimation of population differentiation. Mol. Biol. Evol.

[7] Liu Y.-D., Yang Z.-C., Wu K.-M. (2007). Eight polymorphic microsatellite markers developed in the gree leaf bug, *Lygus lucorum* Meyer-Dür (Hemiptera: Miridae). Mol. Ecol. Notes.

[8] Shrestha R.B., Parajulee M.N., Perera O.P., Scheffler B.E., Densmore L.D. (2007). Characterization of microsatellite loci in the western tarnished plant bug, *Lygus hesperus* Knight (Hemiptera: Miridae). Mol. Ecol. Notes.

[9] Kobayashi T., Sakurai T., Sakakibara M., Watanabe T. (2011). Multiple origins of outbreak populations of a native insect pest in an agro-ecosystem. Bull. Entomol. Res.

[10] Risterucci A.-M., Grivet L., N’Goran J.A.K., Pieretti I., Flament M.H., Lanaud C. (2000). A high density linkage map of *Theobroma cacao* L. Theor. Appl. Genet.

[11] Billote N., Lagoda P.J.L., Risterucci A.-M., Baurens F.-C. (1999). Microsatellite-enriched libraries: applied methodology for the development of SSR markers in tropical crops. Fruits.

[12] Dereeper A., Argout X., Billot C., Rami J.-F., Ruiz M. (2007). SAT, a flexible and optimized Web application for SSR marker development. BMC Bioinforma.

[13] Rozen S., Skaletsky H.J., Krawetz S., Misener S. (2000). Primer3 on the WWW for General Users and for Biologist Programmers. Bioinformatics Methods and Protocols: Methods in Molecular Biology.

[14] Don R.H., Cox P.T., Wainwright B.J., Baker K., Mattick J.S. (1991). “Touchdown” PCR to circumvent spurious priming during gene amplification. Nucleic Acids Res.

[15] Piry S., Alapetite A., Cornuet J.M., Paetkau D., Baudouin L., Estoup A. (2004). GENECLASS2: A software for genetic assignment and first-generation migrant detection. J. Hered.

[16] Rousset F. (2008). Genepop’007: A complete reimplementation of the Genepop software for Windows and Linux. Mol. Ecol. Resour.

[17] Rice W.R. (1989). Analysing tables of statistical tests. Evolution.

[18] Van Oosterhout C., Hutchinson W.F., Wills D.P.M., Shipley P. (2004). Micro-checker: Software for identifying and correcting genotyping errors in microsatellite data. Mol. Ecol. Notes.

[19] Latip S.N.H., Muhamad R., Manjeri G., Tan S.G. (2010). Development of microsatellite markers for *Helopeltis theivora* Waterhouse (Hemiptera: Miridae). Afr. J. Biotechnol.

